# Evaluation of Peruvian Government Interventions to Reduce Childhood Anemia

**DOI:** 10.5334/aogh.2896

**Published:** 2020-08-13

**Authors:** Axel J. Berky, Emily Robie, Ernesto J. Ortiz, Joel N. Meyer, Heileen Hsu-Kim, William K. Pan

**Affiliations:** 1 Nicholas School of the Environment, Duke University, Durham, NC, US; 2 Duke Global Health Institute, Duke University, NC, US; 3 Duke Global Health Innovation Center, Duke University, Durham, NC, US; 4 Department of Civil and Environmental Engineering, Pratt School of Engineering, Duke University, Durham, NC, US

## Abstract

**Background:**

In Peru, anemia has been a persistent health problem that is known to lead to irreversible cognitive and developmental deficits in children. The Peruvian government has recently made anemia a primary health concern by passing legislation in 2017 that makes anemia an intersectoral priority. This new legislation fortifies previous programs while creating new programs that target specific age groups.

**Objectives:**

Evaluate the effectiveness of government programs in Madre de Dios, Peru to reduce anemia prevalence and increase hemoglobin levels among children ages 2–11 years old.

**Methods:**

Propensity scores are used to match 688 children enrolled in 2018, after the legislation, and 2,140 children enrolled in previous studies our team conducted in the region between 2014 and 2017, based on sex, age (years), intervention status (prior/post), community income, presence of a health post in the community (yes/no), community type (indigenous, non-indigenous rural, non-indigenous urban) and road access (fraction of the number of months out of the year with road access). A pseudo matched case-control analysis to evaluate changes in anemia prevalence and hemoglobin was conducted using t-tests and multivariate models. Program effectiveness is evaluated overall, by age groups (2–4, 5–7 and 8–11 years old), and community type (indigenous vs. urban).

**Findings:**

The adjusted odds ratio indicated lower odds of anemia (OR = 0.31, 95%CI 0.17–0.54) for children exposed to the anemia prevention programs vs. those not exposed. The effect was not significantly different across age groups; however, the intervention effects significantly differed by community type among children 8–11 years old, with urban children less likely to benefit from anemia interventions (OR = 0.69, 95% CI 0.38–1.25) compared to indigenous children (OR = 0.21, 95% CI 0.08–0.56).

**Conclusion:**

Government programs to reduce anemia in Madre de Dios were found to be associated with reduced anemia prevalence in the study communities. However, the lack of program monitoring precludes the attribution of anemia decline to specific interventions or program components. In addition, regional anemia prevalence remains high according to the 2019 Demographic and Health Survey, suggesting impaired population impact. Program monitoring and evaluation is a key component of health interventions to improve program implementation effectiveness.

## Introduction

Anemia affects an estimated 1.62 billion people globally, including approximately 47% of pre-school age children (293 million) and 25% of school-age children (305 million) [1]. Anemia is a multifactorial condition characterized by abnormal red blood cells, resulting in poor oxygenation of the body and can lead to irreversible cognitive and developmental deficits in children, increased maternal and child morbidity and mortality, and decreased work productivity in adult populations [2,3,4]. While early treatment can reduce negative health impacts, effects of anemia may be irreversible particularly when experienced during critical developmental periods [5,6].

In Peru, anemia is a public health problem, with 34% of children 6–59 months, and 21% of women 15–49 years of age classified as anemic [7]. Significant regional disparities exist as rural children have a higher rate of anemia compared to urban children (53% vs. 40% prevalence, respectively), and regions of the Amazon have significantly higher rates of anemia compared to metropolitan Lima (54% vs. 33% in children 6–35 months) [7,8]. In Peru’s southern Amazon region of Madre de Dios, Demographic and Health Surveys (ENDES, *La Encuesta Demográfica y de Salud Familiar*) conducted annually since 2004, have consistently estimated anemia prevalence above 40% in children under 5 years (except in 2009, estimated to be 39.4%), ranging between 41% and 51% between 2013 and 2019 (Supplemental Table 1). ENDES corroborates data collected by this research team, which found high anemia prevalence in children under 12 and disproportionately higher in native communities compared to non-native communities [9,10].

In response to this public health problem, the Peruvian government has enacted several interventions to improve anemia outcomes nationally. In 2017, 3 Supreme Decrees, 3 Ministerial Resolutions and 2 Executive Resolutions were enacted at the national level, all pertaining to improvements on child and maternal nutrition and anemia prevention (Supplemental Table 2) [8,11]. Nationwide, there are 8 programs to prevent and decrease anemia, which include screening, surveillance and personalized treatment plans for anemic children 6–36 months, promotion of nutritionally dense foods at education centers, and a deworming program for children 2–17 years of age. These programs intentionally overlap age ranges to reduce the likelihood of gaps in coverage (Supplemental Table 2) [8].

Unfortunately, monitoring and evaluation of these programs at the community or individual levels is not conducted. Even data from ENDES, which provides GPS locations of communities enrolled for some rounds of data collection, have limited application to evaluate policies given that anemia intervention locations and program details are not systematically recorded. However, given the public health significance of anemia and the resources dedicated to these programs, it is important to understand the success and failures of these efforts.

To address this question, the objective of this study is to evaluate whether changes in anemia prevalence and hemoglobin levels from screening studies conducted by our team prior to and after the 2017 ratification of the associated legislation could be attributed to the new prevention programs in Madre de Dios, Peru. We conducted a case-control study using propensity score matching of children aged 2–11 years old enrolled in four studies between 2014 and 2017 with children enrolled in a study in 2018 from the same communities. Results provide guidance on whether government anemia prevention programs can be successful [9].

## Methods

### a. Study Setting and Design

Madre de Dios is a southern Amazon region of Peru characterized by high biodiversity and low population density (2017 census population 141,070), with 34% of the population indigenous, as designated by the government [12]. Anemia is a major problem in Madre de Dios, averaging 44.8% prevalence over the past 15 years according to ENDES (Supplemental Table 1).

Data from this study are from four surveys conducted prior to the 2017 anemia legislation and an anemia study conducted in 2018 (described below). Although surveys were conducted in the same communities, none of these studies were designed to evaluate effectiveness of anemia programs. Therefore, we use propensity score matching of children aged 2–11 years old enrolled prior to and after the 2017 legislation, which is a method that uses a child’s probability of group assignment to balance the pre and post intervention groups [13]. The method is based on the Rubin counterfactual framework and we exclude children who are not well-matched between groups, reducing the potential for systematic error [14]. Propensity scores were computed by intervention status (prior/post) using covariates for sex, age (years), community income, presence of a health post in the community (yes/no), community type (Urban, Rural or Indigenous) and road access (fraction of the number of months out of the year with road access).

### b. Data Collection

Data on anemia and hemoglobin levels collected prior to 2017 are from four studies by our team: the 2014 Interoceanic Highway and River studies ; the Amarakaeri Reserve Cohort study (2015 baseline and 2016 Follow Up); and the Knowledge, Attitudes and Practices of Mercury Exposure study in 2017 [9,15,16,17,18,19,20]. The design of each of these studies varied slightly, but the data collection instruments were similar and every person living in an enrolled household was offered an anemia test using a Hemocue Hb201+ (HemoCue®), following the same protocols across studies—standardization at the start and midpoint of each study using control blood samples provided by the manufacturer. Comparable data used in the present study collected across all studies (including in 2018) include: individual characteristics (age, sex, weight, height, occupation, and education); household characteristics (monthly income and diet); community type (indigenous, non-indigenous rural, urban); and presence of artisanal and small-scale gold mining in the community (yes/no). Household monthly income questions categorized income into nine groups (1 = >200 Peruvian soles [PEN], 2 = 200–300 PEN, 3 = 300–450 PEN, 4 = 450–600 PEN, 5 = 450–600 PEN, 6 = 600–800 PEN, 7 = 800–1,000 PEN, 8 = 1,000–2,000 PEN and 9 = >2,000 PEN), which were then averaged across the community to determine community income.

Enrollment for each study is described in references provided; briefly, the Interoceanic Highway and River studies were 2-stage cluster samples, with random selection of communities (stage 1) and households (stage 2). Ten urban communities from the Interoceanic Highway and five communities located on the Madre de Dios River confluences were purposefully selected, with a total of 48 and 19 communities selected overall and 310 and 83 households, respectively. The Amarakaeri Reserve Cohort purposefully selected 23 communities as part of an environmental impact assessment, enrolling all households with a woman of child-bearing age (WCBA, 15–49) in 19 communities with fewer than 75 households (and an additional 10% sample of households without a WCBA) and selecting a 50% random sample of households in the four urban communities, resulting in a total of 1,221 household enrolled. Follow-up in 2016 was conducted on 458 of 667 nuclear families (e.g., families consisting of a WCBA, her spouse/partner, and a child under 12), who were randomly selected based on a scoring system using principle component analysis to reflect socioeconomic status. The Knowledge, Attitudes and Practices study in 2017 randomly sampled a subset of households from communities that were enrolled in one of the above studies to evaluate family behavior and beliefs regarding mercury exposure and gold mining. All communities included in this anemia program evaluation (except Tres Islas) has at least two prior hemoglobin assessments (Supplemental Table 3).

**Figure 1:**
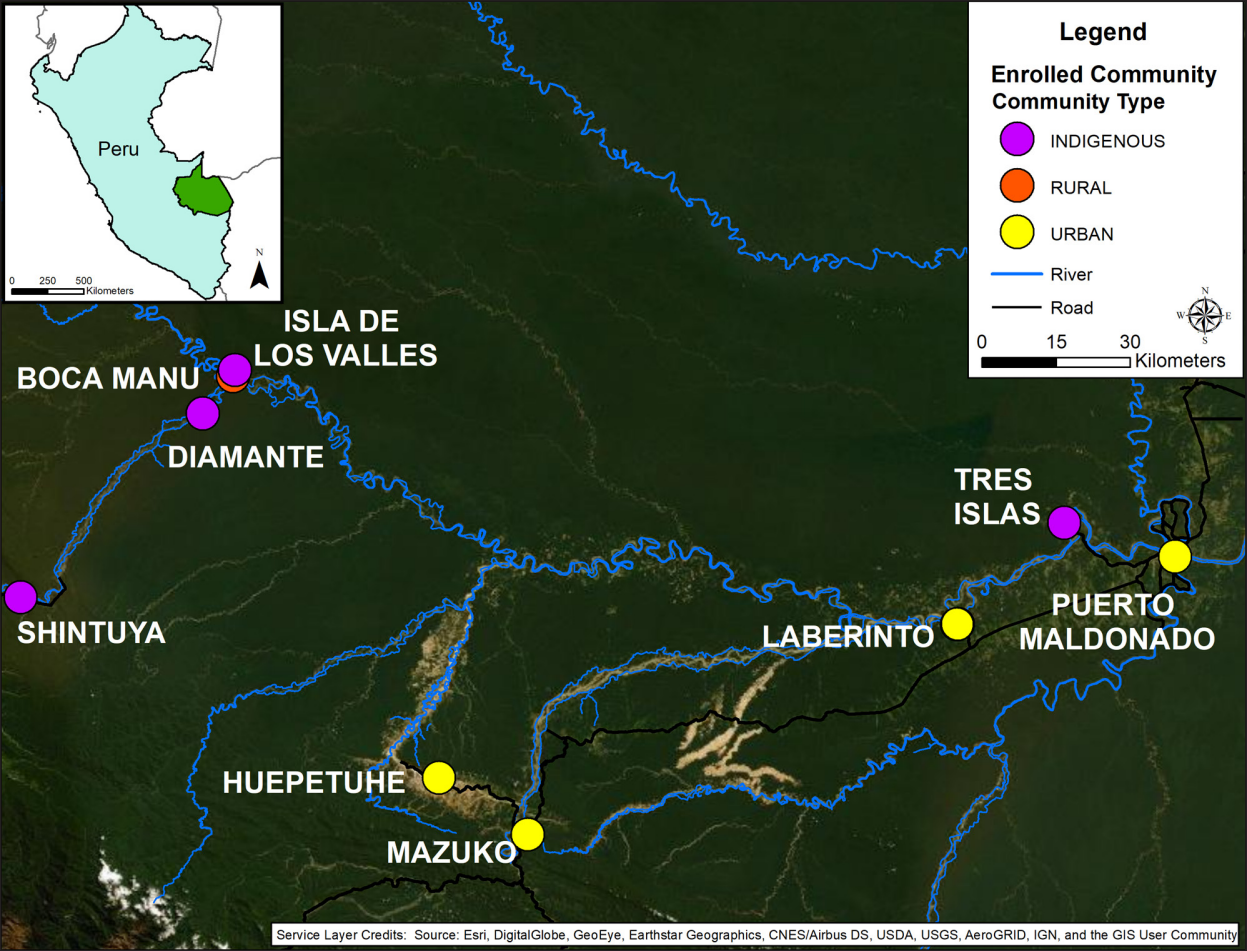
**Project Site Map.** Site map with communities identified as indigenous (purple), non-indigenous rural (orange) or non-indigenous urban (yellow).

In 2018, we designed a study to evaluate anemia etiology and selected communities based on high anemia prevalence from previous studies and their close proximity (within 20km) to artisanal small-scale gold mining [9,16,17,18]. Communities were purposefully selected and classified as either indigenous (n = 4), non-indigenous urban (n = 5) or non-indigenous rural (n=1, defined as communities with fewer than 100 households, per the Peruvian government designation of urban vs rural) (Figure 1). All indigenous communities were also rural; however, we classified them separately as government intervention strategies differ (see Program Evaluation). In each community, we set up anemia screening campaigns in local schools (private and public), after receiving signed consents from parents and verbal consent from the school director. Anemia screening stations were also established in community health posts, allowing for mothers and their children to be screened simultaneously. To ensure an adequate sample size in smaller indigenous and rural communities, we visited individual households by going door to door without a skip pattern, again allowing mothers and their children to be screened simultaneously. The same anemia screening protocols were used as previously described.

### c. Program Evaluation

We obtained information on locations of interventions in Madre de Dios through interviews with the Regional Health Director, appointed heads of the anemia programs, and health workers from the communities enrolled. Although at least 8 programs were established (Supplemental Table 2), we could only confirm existence of intervention activity in our communities and not the specific interventions as specified by law. Thus, we parameterized our model to evaluate the overall effectiveness of the multisectoral approach by comparing anemia prevalence and hemoglobin levels prior to and after the 2017 government legislation.

### d. Statistical Methods

The primary outcomes of interest were anemia classification and hemoglobin level. We used age-specific hemoglobin levels to classify anemia presentation following Peru’s Ministry of Health guidelines. Namely, anemia in children ages 2–4 is defined as having hemoglobin levels below 11.0 g/dL, and in children 5–11 years old below 11.5 g/dL. To further stratify for the purposes of statistical analysis, children under 5 were considered severely anemic at hemoglobin levels below 7.0 g/dL, moderately anemic between 7.0 and 9.9 g/dL, and mildly anemic with levels between 10.0 and 10.9 g/dL. For children 5–11, hemoglobin levels below 8.0 g/dL qualify as severely anemic, 8.0 to 10.9 g/dL as moderately anemic, and 11.0 to 11.4 as mildly anemic, based on WHO criteria [21].

To determine the effect of the government programs started in 2017, children were stratified by age group (2–4, 5–7, and 8–11 years old) to account for attendance at different levels of schooling where food programs and government interventions may vary. Propensity score matching was conducted separately for each age group using the package *MatchIt* in RStudio (V.1.2.5033) for 1-1 matching based on the covariates previously mentioned.

The anemia intervention was evaluated after propensity score matching was completed for all ages combined and for each age group (2–4, 5–7, 8–11) using two approaches. First, t-tests were conducted to compare anemia status and hemoglobin values prior to and post implementation of government programs for each age group. Second, multivariate models were fit to evaluate differences in anemia and hemoglobin, adjusting for age, sex, community income, and community type (indigenous, rural, urban or mining vs. non-mining). Variables remained in the model if their Type III test resulted in a p-value under 0.20, otherwise they were removed individually using a backward-selection procedure. The model for all ages combined excluded community type to produce an overall effect estimate, while the age group models included an interaction to test for differences in intervention effects between urban and indigenous communities (excluding the one non-indigenous rural community). A generalized linear mixed model approach was used; specifically, a logit model to evaluate anemia differences and a linear model for hemoglobin. Inclusion of a community random effect was evaluated by comparing AIC values of nested models that included or excluded the random effect. Community random effects improved model fit for anemia (logit) models for children 2–11, and 5–7, and hemoglobin (linear) models in children ages 2–11 and 5–7.

## Results

In total 207, 248 and 233 children ages 2–4, 5–7, and 8–11 were screened for anemia in 2018. These children were matched 1-1 by age group using propensity scores that drew from 525, 470, and 587 children ages 2–4, 5–7, and 8–11, respectively, that were enrolled in studies conducted between 2014–2017 (Table 1). The majority of anemia identified in this study were considered mild, with few cases of moderate anemia and none classified as severe anemia.

**Table 1:** Mean hemoglobin (g/dL) and anemia status by age group prior and post government legislation passed in 2017.

Ages	Prior (2014–2017)				Post (2018)			

	Mean Hemoglobin	Not Anemic	Anemic	Prevalence (%)	Mean Hemoglobin	Not Anemic	Anemic	Prevalence (%)

2–4 Years Old	11.14	115	92	44	11.69	161	46	22***
5–7 Years Old	11.50	132	116	47	11.94	170	78	31**
8–11 Years Old	11.97	166	67	29	12.53	201	32	14***
All Ages	11.55	413	275	40	12.06	532	156	23***


*** p < 0.0001; ** p < 0.01; * p < 0.05.

**Figure 2:**
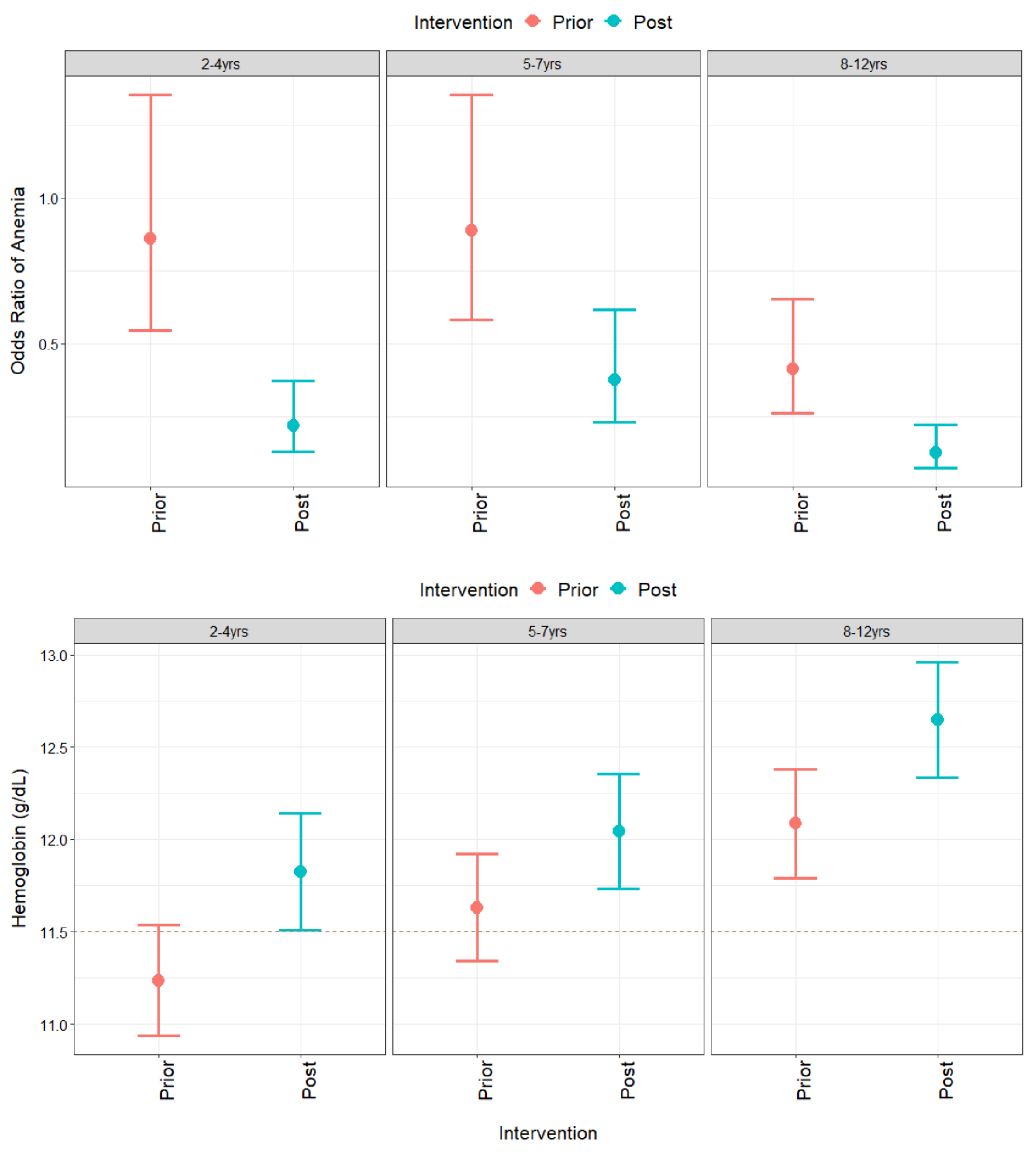
**Model Estimates of Anemia Prevalence and Hemoglobin Levels by Age Group.** Model adjusted intervention effects for the odds ratio of anemia (top panel) and blood hemoglobin levels (bottom panel) with 95% confidence intervals by age group. Model estimates incorporate mean age, household income and other covariates from the model selection procedure (Tables 2–4). Levels prior to (red) and post (blue) the 2017 government legislation are shown along with a dotted line indicating Peru’s Ministry of Health definition of anemia for children 5–11 years old; the value for children ages 2–4 is below 11.0 g/dL (see Method for WHO cut-offs).

Overall anemia prevalence significantly declined in the matched groups from 40% prior to the government legislation to 23% in 2018 (Table 1). Anemia prevalence declined from 44% to 22% in children 2–4 years old (p < 0.0001), from 47% to 31% in children 5–7 years old (p < 0.0001), and from 29% to 14% in children 8–11 years old (p < 0.0001), with equivalent increases in hemoglobin for each age group before and after the intervention (Table 1). After adjusting for age, community income and mining status, model results indicate that children who were exposed to government interventions had a 0.31 (95% CI 0.17–0.54) lower odds of anemia compared to children before the interventions were implemented (Table 2). Although there were nominal differences in the intervention effects by age group, none of these differences were significant (Figure 2). Similarly, the government interventions were associated with a significant 0.52 (95% CI 0.32–0.81) increase in hemoglobin levels in children, which also did not vary significantly by age group according to the interaction test between age group and intervention (Table 3). Note that higher community income and living in a mining community were both associated with lower odds of anemia, but these effects were not significant except for children 2–4 (odds ratio of anemia among children living in a mining community: 0.32 (95% CI, 0.17–0.60). Age-stratified models also showed that older children within each strata has significantly lower odds of anemia compared to younger children, except among children 5–7 years old, which further showed that males had higher risk of anemia and lower hemoglobin levels than females (OR = 1.49, 95% CI 1.01–2.21; Hb = –0.18, 95% CI –0.38–0.02).

**Table 2:** Logit model results for anemic status (Yes/No) with all ages and by age group (2–4, 5–7, and 8–11), rural community excluded.

	All Ages Combined^1^		2–4 year olds^1^		5–7 Year olds^1^		8–11 year olds^1^	

	Odds Ratio	(95% CI)	Odds Ratio	(95% CI)	Odds Ratio	(95% CI)	Odds Ratio	(95% CI)

Intervention: Post (vs. Prior)^2^	0.31	(0.17– 0.54)***	0.42	(0.15–1.12).	0.31	(0.11–0.86)*	0.21	(0.07–0.53)**
Age (Years) Continuous (separate model)			0.71	(0.53–0.95)*	ns		0.71	(0.56–0.88)**
Age^3^ 2–4 year olds	2.09	(1.38–3.18)***						
5–7 year olds	2.16	(1.43–3.26)***						
8–11 year olds	Ref							
Sex: Male (vs. Female)	ns		ns		1.49	(01.01–2.21)*	ns	
Community: URBAN (vs. Native)	ns		1.21	(0.55–2.73)	0.87	(0.32–2.36)	0.23	(0.10–0.45)***
Community Income (PEN)	0.77	(0.51–1.14)	ns		ns		0.49	(0.30–0.78)**
Mining Community: Yes (vs. No)	0.65	(0.33–1.28)	0.32	(0.17–0.60)***	0.75	(0.34–1.66)	ns	
**Interactions**								

Intervention:* 2–4 yrs (vs. 8–11 yrs)	0.83	(0.43–1.59)						
Intervention:* 5–7 yrs (vs. 8–11 yrs)	1.38	(0.74–2.57)						
Intervention* URBAN			0.64	(0.19–2.15)	1.28	(0.32–5.11)	3.28	(1.09–10.99)*


1. The log-odds intercept for each model is, respectively (All Ages to 8–11 year olds): 2.41 (95% CI: 0.18–31.85); 1.52 (95% CI: 0.97–2.41); 1.18 (95% CI: 0.70–1.99); and 1.12 (95% CI: 0.68–1.88). 2. The marginal (population) effects and 95% CIs of the intervention for each age category (All Ages, 2–4, 5–7, 8–11 years) were, respectively: 0.32 (0.21–0.48); 0.34 (0.19–0.60); 0.35 (0.19–0.64); and 0.38 (0.21–0.68). 3. Age is categorical for the All Ages Combined model, but continuous for the age-specific models. Age is centered at 3, 6, and 9 years for the 2–4, 5–7, and 8–11 year old models, respectively. *** p < 0.0001; ** p < 0.01; * p < 0.05; . p < 0.10.

**Table 3:** Linear model results for hemoglobin (g/dL) with all ages and and by age group (2–4, 5–7, and 8–11), rural community excluded.

	All Ages Combined^1^		2–4 year olds^1^		5–7 Year olds^1^		8–11 year olds^1^	

	Beta	(95% CI)	Beta	(95% CI)	Beta	(95% CI)	Beta	(95% CI)

Intervention: Post (vs. Prior)^2^	0.56	0.(32–0.81)***	0.89	(0.45–01.32)***	0.21	(–0.36–0.78)	0.90	(0.52–1.29)***
Age (Years)			0.26	(0.14–0.39)***	ns		0.21	(0.12–0.31)***
Age^3^ 2–4 year olds	–0.85	(–1.06––0.64)***						
5–7 year olds	–0.45	(–0.66––0.25)***						
8–11 year olds	REF							
Sex: Male (vs. Female)	Ns		ns		–0.18	(–0.38–0.02).	ns	
Community: URBAN (vs. Native)	ns		ns		0.37	(–0.47–1.21)	0.65	(0.31–0.99)***
Community Income (PEN)	ns		ns		ns		0.24	(0.03–0.45)*
Mining Community: Yes (vs. No)	0.38	(–0.10–0.87)	0.36	(0.11–0.61)**	ns		ns	
**Interactions**								

Intervention:* 2–4 yrs (vs. 8–11 yrs)	0.02	(–0.27–0.32)						
Intervention:* 5–7 yrs (vs. 8–11 yrs)	–0.15	(–0.43–0.13)						
Intervention* URBAN			–0.49	(–1.01–0.03).	0.3.	(–0.41–1.01)	–0.56	(–1.02––0.11)*


1. The intercept for each model is respectively (All Ages to 8–11 years old): 11.89 (95% CI: 11.59–12.21); 10.73 (95% CI: 10.52–10.94); 11.25 (95% CI: 10.77–11.74); 9.97.60 (95% CI: 8.55–11.38). 2. The marginal (population) effects and 95% CIs of the intervention for each age category (All Ages, 2–4, 5–7, 8–11 years) were, respectively: 0.52 (0.33–0.71); 0.65 (0.40–0.90); 0.38 (0.02–0.74); and 0.62 (0.39–0.85). 3. Age is categorical for the All Ages Combined model, but continuous for the age-specific models. Age is centered at 3, 6, and 9 years for the 2–4, 5–7, and 8–11 year old models, respectively. *** p < 0.0001; ** p < 0.01; * p < 0.05; . p < 0.10.

**Figure 3:**
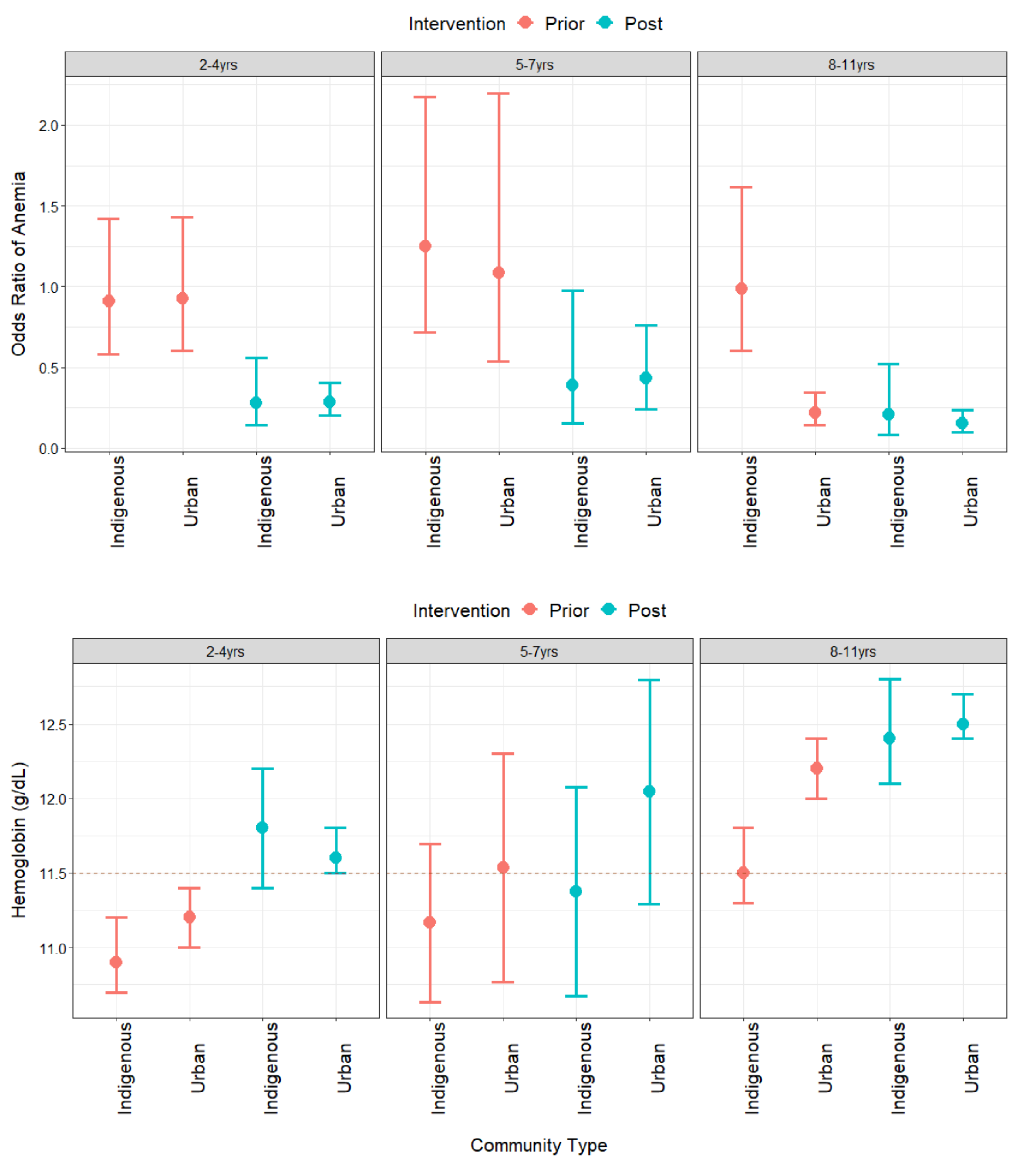
**Model Estimates of Anemia Prevalence and Hemoglobin Levels by Community Type.** Model adjusted intervention effects for the odds ratio of anemia (top panel) and blood hemoglobin levels (bottom panel) by community type and age group (excluding rural communities). Levels prior to (red) and post (blue) the 2017 government legislation are shown along with a dotted line indicating Peru’s Ministry of Health definition of anemia for children 5–11 years old; the value for children ages 2–4 is below 11.0 g/dL (see Method for WHO cut-offs).

Anemia prevalence in 2018 varied by community type, with 22%, 23%, and 5.9% of children classified as anemic in indigenous, urban, and rural communities, respectively, a decline from 53%, 34%, and 25% prior to 2018 (Supplemental Table 3). Stratifying by age group, we evaluated intervention effects in indigenous versus urban communities (excluding the one rural community). Models confirmed no differences in the effect sizes of the intervention by age group and found no difference in the intervention effect between urban and indigenous communities for children 2–4 and 5–7 years old (Tables 2 and 3); however, the anemia model for 5–7 year olds indicated a nominally significant intervention difference prior to inclusion of the random community effect (0.857 lower log odds of anemia in indigenous vs. urban communities following intervention, p=0.0843, model not shown). Among 8–11 year olds, anemia intervention programs had a larger effect among children living in indigenous communities (0.21 odds of anemia, 95% CI 0.08–0.56) following interventions compared to children living in urban areas (0.69, 95% CI 0.38–1.25) (p = 0.0419, Table 2, Figure 3). Similarly, for hemoglobin, intervention by community (indigenous vs. urban) was not significant, except for ages 8–11, in which hemoglobin levels increased an additional 0.56g/dL in indigenous communities (Table 3).

## Discussion

This study illustrates that the odds of anemia reduced by nearly 70% and hemoglobin levels increased by 0.56g/dL after the implementation of the 2017 anemia legislation in Madre de Dios, Peru. These effects were found to be consistent across all age groups ranging from 2 to 11 years old. In addition, effect sizes were consistent across community types, demonstrating a broad impact in the study communities that reduced previous health disparities between urban and indigenous communities (Figures 2 and 3). However, we did find differences in intervention effects between urban and indigenous communities among children ages 8–11, in which the intervention was more effective for indigenous communities as anemia prevalence (and average hemoglobin) was already low (high) in urban communities prior to 2018 (22%, Hb=12.1g/dL). This observational study supports the success of recently implemented anemia programs in the study communities.

Our results further highlight that communities with higher income and that have active gold mining, which also tend be wealthier, were associated with lower anemia risk (higher hemoglobin). We observed larger intervention effects in poorer communities compared to wealthier ones, although this is due to wealthier communities having less anemia (data not shown). Other studies conducted in Peru have found a similar association [22,23].

While economic differences may partially explain higher anemia prevalence in indigenous communities, additional efforts are needed to assess why anemia rates in indigenous communities remain higher than those in non-indigenous communities. Recent studies have shown indigenous communities to have significantly higher levels of mercury that often surpass World Health Organization’s guidelines, and have been associated with lower hemoglobin levels [9,20,24]. Lack of effective medical access, poor nutrition, enteric disease or other unknown factors may also play a role. To better understand the etiology of anemia in indigenous communities, future studies must identify the proportion of anemia cases due to iron deficiency, enteric disease, vitamin A deficiency as well as other etiologies to strengthen government programs. As the Peruvian Ministry of Health continues to expand programs to combat anemia, the effect of community context must be better understood to evaluate and design anemia programs.

Before the national legislation was signed in 2017, effective anemia reduction had been observed at the national level. Demographic and Health Surveys (ENDES) data from 2000 report 50% of children under 5 with anemia, which fell to 33% in 2012 but has remained at that level through 2018 [25]. These national trends primarily reflect urban communities that experienced a 48% decline in anemia during a period of economic growth (1996–2011), while no decline was identified in rural communities [25]. Anemia levels in Madre de Dios, an Amazon region with the smallest population nationwide, are likely not benefiting from national economic growth.

Although our results indicate that the anemia intervention programs can be successful, ENDES data from Madre de Dios in 2018 and 2019 indicate high anemia prevalence (42.4% among children under 5). As ENDES is a population level survey, it is reasonable to assume that the anemia prevention programs are not achieving the success as measured by this study. This discrepancy could be related to study limitations, limitations in ENDES, or inadequate program rollout to achieve a population level impact. Study limitations are discussed below, but it is important to note that ENDES, like the original studies from which this study is predicated, is not designed to evaluate government interventions. Without a proper design to measure propensity to be exposed to an intervention and matched to comparable children, simple prevalence estimates from ENDES cannot be used for inference. Assuming both the current study and ENDES accurately measure program impact and population prevalence, respectively, we might conclude that program implementation and rollout for the region as a whole has failed. Regardless of these potential discrepancies, it is undeniable that the lack of program monitoring and evaluation is a significant impediment to achieving the intended goals of the anemia legislation.

The study has important limitations. First, the study is not a population sample, rather, it focuses on the southern and southwestern regions of Madre de Dios and does not incorporate historical or present anemia levels in the northern part of the department. Further, anemia screening in 2018 occurred mainly in schools and health centers, which may not be representative of all community members. Lastly, most of the children screened in 2018 were from indigenous (n = 200) and urban (n = 1,002) communities, while only 22 children were screened from one rural community, precluding our ability to evaluate the effectiveness of the interventions in rural (non-indigenous) communities.

Regardless of study limitations, we use a valid approach to measure the effectiveness of intervention programs implemented in communities we visited in 2018, concluding that these programs can reduce anemia by up to 70%. However, the lack of program monitoring, particularly in a decentralized system, significantly impairs that ability to evaluate and modify programs to improve effectiveness. This study indicates that community and regional scale monitoring criteria, such as program implementation dates, food types distributed, and number of children receiving assistance needs to be immediately implemented. The lack of monitoring and evaluation criteria to assess government programs at the national and regional level prevents a more definitive, detailed analysis of anemia prevalence and the effectiveness of government programs.

## Conclusion

Efforts to reduce anemia prevalence have continued to be paramount as another Supreme Decree was ratified on July 2, 2018, implementing a multisectoral approach in reducing levels of anemia nationwide that incorporated 14 different governmental ministries.^8^ As the Peruvian government implements policies to address anemia nationwide, further evaluation on the policy and program implementation are necessary to identify vulnerable populations and potential mechanisms to improve program effectiveness.

## Data Accessibility Statement

The datasets generated and/or analyzed during the current study are not publicly available due continuing publications but are available from the corresponding author on reasonable request.

## Additional Files

The additional files for this article can be found as follows:

10.5334/aogh.2896.s1 Supplemental Table 1. Anemia prevalence in Madre de Dios, Peru for children 6–59 months according to DHS and ENDES surveys conducted between 2004–2019.

10.5334/aogh.2896.s2 Supplemental Table 2. Ratified Peruvian legislation to implement anemia prevention programs in 2017.

10.5334/aogh.2896.s3 Supplemental Table 3. Anemia prevalence in study communities across five studies: Interoceanic Highway (IOH) and River Studies in 2014; Amarakaeri Reserve Cohort (ARC) in 2015, Amarakaeri Reserve Cohort Follow Up in 2016, Knowledge, Attitudes and Practices (KAP) in 2017 and Etiology of Anemia in 2018. Anemia prevalence in red were inferred from matched communities with similar characteristics such as community type and located within 10 kilometers.
