# Supplementary material for: Evaluation of Peruvian Government Interventions to Reduce Childhood Anemia
Source: Ann Glob Health. 2020 Aug 13;86(1):98. doi: 10.5334/aogh.2896 (PMC7427686; doi:10.5334/aogh.2896)
Supplement: Supplemental Table 1. — Anemia prevalence in Madre de Dios, Peru for children 6–59 months according to DHS and ENDES surveys conducted between 2004–2019. [file agh-86-1-2896-s1.pdf]

Supplemental Table 1. Anemia prevalence in Madre de Dios, Peru for children 6–59 months according to DHS and ENDES surveys conducted between 2004 – 2019.

Year	Anemia Prevalence in Madre de Dios(%)	National Anemia Prevalence in Peru
2004-2006[26]	57	46.2
2007-2008[27]	48.1	42.4
2009	39.4	37.2
2010	44.7	37.7
2011	41.8	30.7
2012	39.9	32.9
2013	48.2	34.0
2014	51.3	35.6
2015[28]	44.3	32.6
2016[29]	42.9	33.3
2017[30]	44.9	34.1
2018[31]	41.6	32.8
2019[32]	43.2	29.5

Data are from the *Peru Encuesta Demografia y de Salud Familiar* (ENDES). Data collection from 2004 to 2014 was conducted by the Measure DHS, ORC Macro and the Instituto Nacional de Estadistica e Informatica (INEI). Surveys since 2015 were conducted by INEI. ENDES and the present study have the same methods, field equipment, and cutoffs for anemia.

References

26. **Instituto Nacional de Estadística e Informática (INEI)**. Encuesta Demográfica y de Salud FamiliarENDES Continua 2004-2006. Informatica INdEE, ed. Peru. 2007.
27. **Instituto Nacional de Estadística e Informática (INEI)**. Informe de la Encuesta Demográfica y de Salud Familiar - ENDES Continua 2007-2008. Infomatica INEE, ed. Peru. 2008.
28. **Instituto Nacional de Estadística e Informática (INEI)**. Perú: Encuesta Demográfica y de Salud Familiar 2015 - Nacional y Departamental. Informatica INdEE, ed. Peru. 2016.
29. **Instituto Nacional de Estadística e Informática (INEI)**. Perú: Encuesta Demográfica y de Salud Familiar 2016 - Nacional y Regional. Informatica INEE, ed. Peru. 2017.
30. **Instituto Nacional de Estadística e Informática (INEI)**. Perú: Encuesta Demográfica y de Salud Familiar 2017 - Nacional y Regional. Informatica INEE, ed. Peru. 2018.
31. **Instituto Nacional de Estadística e Informática (INEI)**. Perú: Encuesta Demográfica y de Salud Familiar 2018 - Nacional y Departamental". Informatica INdEE, ed. Peru. 2019.
32. **Instituto Nacional de Estadística e Informática (INEI)**. Perú: Encuesta Demográfica y de Salud Familiar 2019 - Nacional y Departamental. Informatica INdEE, ed. Peru. 2020.
