# Supplementary material for: Evaluation of Peruvian Government Interventions to Reduce Childhood Anemia
Source: Ann Glob Health. 2020 Aug 13;86(1):98. doi: 10.5334/aogh.2896 (PMC7427686; doi:10.5334/aogh.2896)
Supplement: Supplemental Table 2. — Ratified Peruvian legislation to implement anemia prevention programs in 2017. [file agh-86-1-2896-s2.pdf]

Supplemental Table 2. Ratified Peruvian legislation to implement anemia prevention programs in 2017.

Signed Decrees in 2017 by the Peruvian Federal Government	Objective	Link
Resolución Ministerial N° 506-2017-MINSA	• Anemia tests for children 6–36 months and initiation of the national program Cuna Mas by MIDIS. Anemic children will be given treatment and a treatment plan will be established that will last for 6 months. Health officials will inform parents how to give supplements, proper nutrition and causes of anemia.	https://www.gob.pe/institucion/minsa/normas-legales/189252-506-2017-minsa
Resolución Ministerial N° 249-2017/MINSA	• Contribute at the national level, to the reduction and monitoring of maternal and infant anemia and chronic malnutrition through the strengthening of effective intersectoral interventions.<br>• Preventative iron supplements at 4 months. In children over 6 months: promote iron rich food, iron supplements, offer food fortified in iron, at age two provide antiparasitic tablets. Promote overlapping interventions to improve health outcomes.<br>• Establishes 15 activities to promote better health that consist of deworming program (2–17 years old) at school, household visits, vitamin A and iron supplements and various types of trainings such as food preparation and hand washing.	https://cdn.www.gob.pe/uploads/document/file/190343/189839_RM_249-2017-MINSA.PDF20180823-24725-1d4msjm.PDF
Decreto Supremo N° 005-2017-MC	• Fundamental human right and dignity of the people to reduce social differences through the eradication of poverty and extreme poverty and the access to land in favor of indigenous communities.	https://cdn.www.gob.pe/uploads/document/file/211260/ds005-2017-mc23102017100229.pdf
Decreto Supremo N° 016-2017-PRODUCE	• Continuation of the national program "A Comer Pescado" until 2021. The program aims to increase the per capita consumption of seafood and other aquatic organisms such as freshwater fish to improve diet.	https://cdn.www.gob.pe/uploads/document/file/135001/79283_1.pdf

Decreto Supremo N° 017-2017-SA	• Approves Law 30021, the law of the Promotion of Healthy Alimentation, which establishes the growth and adequate development of individuals through education and the implementation of kiosks and diners in basic educational institutions.	http://repositorio.indecopi.gob.pe/bitstream/handle/11724/5731/DS.017-2017-SA.pdf?sequence=1&isAllowed=y
Resolución Ministerial Nº 250-2017/MINSA	• Approves the Technical Norm for the management and prevention of anemia in children, adolescents, pregnant women and infants. Entitle the Office of Strategic Interventions in Public Health with the diffusion, monitoring, supervision and evaluation of the implementation of the Technical Health Norm.<br>• Contribute to the development and well-being of children, adolescents, pregnant women and infants in integral healthcare.<br>• Provides clinical criteria for diagnosis and methods of treatment and prevention. For example, anemia testing at 4 months of age and preventative supplementation of iron at 6 months.	https://cdn.www.gob.pe/uploads/document/file/190345/189840_RM_250-2017-MINSA.PDF20180823-24725-1rsx1wh.PDF
Resolución de Dirección Ejecutiva N° 130-2017-MIDIS-PNADP-DE	• Modified the rules and regulations for the affiliation and permanence of households in the National Program “Juntos” that focuses on strategies against chronic childhood anemia and malnutrition.	https://busquedas.elperuano.pe/normaslegales/aprueban-reglas-adicionales-para-la-afiliacion-y-permanenci-resolucion-no-114-2017midispnadp-de-1549246-1/
Resolución Ministerial Nº 112-2017/MIDIS	• Plan to reduce chronic infant malnutrition in children younger than 36 months through the strengthening of intragovernmental actions, social program interventions and the establishment of incentives that are promoted by the Ministry of Development and Social Inclusion.	https://cdn.www.gob.pe/uploads/document/file/15785/RM_112_2017MIDIS.pdf
