# Supplementary material for: Evaluation of Peruvian Government Interventions to Reduce Childhood Anemia
Source: Ann Glob Health. 2020 Aug 13;86(1):98. doi: 10.5334/aogh.2896 (PMC7427686; doi:10.5334/aogh.2896)
Supplement: Supplemental Table 3. — Anemia prevalence in study communities across five studies: Interoceanic Highway (IOH) and River Studies in 2014; Amarakaeri Reserve Cohort (ARC) in 2015, Amarakaeri Reserve Cohort Follow Up in 2016, Knowledge, Attitudes and Practices (KAP) in 2017 and Etiology of Anemia in 2018. Anemia prevalence in red were inferred from matched communities with similar characteristics such as community type and located within 10 kilometers. [file agh-86-1-2896-s3.pdf]

Supplemental Table 3. Anemia prevalence in study communities across five studies: Interoceanic Highway (IOH) and River Studies in 2014; Amarakaeri Reserve Cohort (ARC) in 2015, Amarakaeri Reserve Cohort Follow Up in 2016, Knowledge, Attitudes and Practices (KAP) in 2017 and Etiology of Anemia in 2018. Anemia prevalence in red were inferred from matched communities with similar characteristics such as community type and located within 10 kilometers.

Unmatched Anemia Prevalence (%) in Study Communities from 2014 – 2018

		Study (Year)				
Community Name	Community Type	IOH and River Studies (2014)	ARC (2015)	ARC Follow Up (2016)	KAP (2017)	Etiology of Anemia (2018)
Boca Manu	Rural	0.0	23.0	25.6		5.9
Diamante	Indigenous		35.3	41.9		29.8
Puerto Maldonado	Urban	34.0			51.8	33.1
Huepetuhe	Urban		29.6	49.3	31.3	15.7
Isla de los Valles	Indigenous		28.6	60.0		33.3
Laberinto	Urban	18.8			33.3	26.2
Mazuko	Urban	23.1	23.0			25.5
Shintuya	Indigenous		32.3	0.0		16.1
Tres Islas	Indigenous	44.4	60.0	64.0		19.4
